# Gold Containing Antimony

**Published:** 1860-04

**Authors:** 


### EDITORIAL DEPARTMENT.

*Gold Containing Antimony*—Mr. Warrington has recently called the attention of the Chemical Society of London, to the existence of an alloy of gold very troublesome to those who work that precious metal. He first met with it in a bar of Australian gold, said to have been obtained by the quartz-crushing process, which looked very well on the outside, but when broken, proved to be grayish yellow, and crystalline within. It was extremely brittle and utterly unfit for rolling or mixing with other gold, to which it communicated its own bad qualities. Much of this sort of gold had been brought in, and the royal mint had returned to the Bank of England, as unfit for the purposes of coinage, 47,000 ounces of it. The following tables express the composition of two samples of this gold, No. I, representing the constitution of the original bar, and No. II, that of a second specimen, both examined by Mr. Warrington.

| | No. I. | No. II. |
|---|---|---|
| Gold, | 92.40 | 93.80 |
| Silver, | 4.60 | 2.20 |
| Antimony, | Trace. | 2.28 |
| Tin, | 2.00 | 1.40 |
| Arsenic | | Trace. |
| Copper, | 0.75 | do. |
| Loss, | 0.25 | 0.32 |
| | 100.00 | 100.00 |

Mr. Warrington is inclined to attribute the presence of antimony to an attempt to refine the gold by the use of sulphuret of antimony, which is sure to contaminate the precious metal unless great precaution is used. The tin may have been introduced at the mines, as a good deal of stream tin comes from Australia.

Mr. W. suggested a very simple and cheap expedient for getting rid of these troublesome metals. It is to oxydize them at the expense of some metallic oxyd, having, at high temperatures, a weaker affinity for oxygen than the metals in question. Nitrate of potash will not answer the purpose owing to its low specific gravity. Oxyd of copper is the substance preferred by our author. The gold to be refined is mixed with ten per cent. of this oxyd, and a small quantity of borax, and kept in fusion with them for half an hour. The result is a perfectly malleable gold, containing a small per centage of copper. The proportion of the oxyd of copper must, of course, vary with the quantity of oxydizable metal to be removed. The tyro in chemistry must bear in mind another practical fact, which is, that black-lead crucibles cannot be used for this method of refining, as the oxyd of copper would be reduced by the carbon of the crucible, while the antimony would not be affected by it.

During the discussion which followed the reading of this paper, it was mentioned that the gold made from sweepings of book binders, &c., was frequently contaminated by antimony in consequence of the accidental admixture of loose type. It is well for dentists to be on the look out for these unmalleable qualities of gold.

